# Incidence of Sarcoma Histotypes and Molecular Subtypes in a Prospective Epidemiological Study with Central Pathology Review and Molecular Testing

**DOI:** 10.1371/journal.pone.0020294

**Published:** 2011-08-03

**Authors:** Françoise Ducimetière, Antoine Lurkin, Dominique Ranchère-Vince, Anne-Valérie Decouvelaere, Michel Péoc'h, Luc Istier, Philippe Chalabreysse, Christine Muller, Laurent Alberti, Pierre-Paul Bringuier, Jean-Yves Scoazec, Anne-Marie Schott, Christophe Bergeron, Dominic Cellier, Jean-Yves Blay, Isabelle Ray-Coquard

**Affiliations:** 1 Université de Lyon, Cancer Centre Leon Berard, EA 4129, Lyon, France; 2 Université de Lyon, Cancer Centre Leon Berard, Department of Pathology, Lyon, France; 3 Université de Saint-Etienne, Centre Hospitalier Universitaire, Department of Pathology, Saint-Etienne, France; 4 Laboratory of Pathology, Pringy, France; 5 Laboratory of Pathology, Cypath, Lyon, France; 6 Laboratory of Pathology, Grenoble, France; 7 Université de Lyon, Cancer Centre Leon Berard, INSERM U590, Lyon, France; 8 Université de Lyon, Hospices Civils de Lyon, Hôpital Edouard Herriot, Department of Pathology, Lyon, France; 9 Université de Lyon, Hospices Civils de Lyon, Pôle IMER, Lyon, France; 10 Institut d'Hématologie et d'Oncologie Pédiatrique, Department of Paediatrics, Lyon, France; 11 Merck Serono, Scientific Relation, Lyon, France; City of Hope National Medical Center and Beckman Research Institute, United States of America

## Abstract

**Background:**

The exact overall incidence of sarcoma and sarcoma subtypes is not known. The objective of the present population-based study was to determine this incidence in a European region (Rhone-Alpes) of six million inhabitants, based on a central pathological review of the cases.

**Methodology/Principal Findings:**

From March 2005 to February 2007, pathology reports and tumor blocks were prospectively collected from the 158 pathologists of the Rhone-Alpes region. All diagnosed or suspected cases of sarcoma were collected, reviewed centrally, examined for molecular alterations and classified according to the 2002 World Health Organization classification. Of the 1287 patients screened during the study period, 748 met the criteria for inclusion in the study. The overall crude and world age-standardized incidence rates were respectively 6.2 and 4.8 per 100,000/year. Incidence rates for soft tissue, visceral and bone sarcomas were respectively 3.6, 2.0 and 0.6 per 100,000. The most frequent histological subtypes were gastrointestinal stromal tumor (18%; 1.1/100,000), unclassified sarcoma (16%; 1/100,000), liposarcoma (15%; 0.9/100,000) and leiomyosarcoma (11%; 0.7/100,000).

**Conclusions/Significance:**

The observed incidence of sarcomas was higher than expected. This study is the first detailed investigation of the crude incidence of histological and molecular subtypes of sarcomas.

## Introduction

Sarcomas are a heterogeneous group of rare malignant tumors of connective tissues, capable of differentiation into many different cell types such as connective tissues (lipocytes, fibrous supporting structures, muscle, etc.), visceral tissues and bone. These tumors can occur in almost any anatomic site, although they are reported to be more frequent in the extremities [1]. In most comprehensive reviews, soft tissue sarcomas (STS) are reported to account for respectively 0.7–1% and 4–8% of all adult and pediatric malignant tumors, and bone sarcomas for respectively 0.2% and 5% [2]–[4]. More than fifty histological subtypes were described in the classification of the World Health Organization (WHO) updated in 2002 [5], and further refinements have been made since with the publication of molecular classification studies identifying distinct molecular subtypes [6].

The exact overall incidence of sarcomas is unknown and the incidence of the different histological and molecular subtypes has not been determined precisely [7]. Most studies consider adults and children, or soft tissue and bone sarcomas separately. Results of population studies indicate that the incidence of sarcomas may actually be underestimated, possibly because of diagnostic confusion with carcinomas of the same organ. Recent reports of a 1.1–1.5/100,000/year incidence of gastrointestinal stromal tumors (GIST) [8] suggest that the overall incidence rates of 1 to 3/100,000/year generally reported are likely underestimated [9]. In addition, sarcomas encompass a wide variety of histotypes and molecular subtypes and are categorized in rapidly evolving phenotypic and molecular subgroups now used for sarcoma diagnosis with a growing impact on the management of patients [10].

The aim of the present study was to describe the overall incidence of sarcoma and the precise incidence of the different histological and molecular subtypes in a given European region.

## Materials and Methods

This prospective study involves the exhaustive collection of all incident cases of sarcoma in the French Rhone-Alpes (RA) region (6,021,352 inhabitants, 2006 census, 10% of the French population) over a two-year period (March 2005 to March 2007).

### Inclusion and exclusion criteria

All Rhone-Alpes patients with a diagnosis of primary sarcoma made by any public or private pathology laboratory of the region between March 1, 2005 and February 28, 2007, were included in the study. All subtypes of sarcoma were eligible: soft tissue, bone, or visceral tumors (GIST, gynecological sarcomas), Kaposi sarcomas, etc. Relapsing patients were excluded. Other exclusion criteria were date of initial diagnosis outside the registration period, patients living outside the region at the date of diagnosis (selected based on postal code), and no evidence of sarcoma upon histological review. All patients' medical records were reviewed for exclusion criteria.

### Data collection

**Figure 1:**
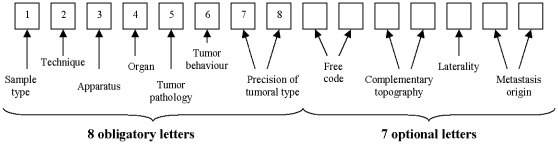
The French ADICAP coding system (http://www.adicap.asso.fr/).

All 43 pathology laboratories (N = 158 pathologists) of the RA region agreed to participate in the study. They prospectively reported all newly suspected cases and provided paraffin-embedded blocks for systematic review. Onsite monitoring visits were conducted to ensure exhaustiveness of registration. The pathologists were given financial compensation for each case they reported. Two pathologists of the comprehensive cancer centre, Dominique Ranchère-Vince (DRV) and Anne-Valerie Decouvelaere (AVD), established a list of ADICAP codes corresponding to subtypes of sarcoma to be used by the pathology laboratories for patient registration. The French ADICAP coding system is an accurate mnemonic alphanumeric thesaurus, currently in use in France to classify histological subtypes and is equivalent to the International Classification of Diseases for Oncology (ICDO) codes (Figure 1). Pathologists were also required to report tumors with uncertain diagnosis or ambiguous subtype, so that sarcomas initially misdiagnosed as benign lesions or categorized into different subsets (carcinomas) would not be missed.

### Control of exhaustiveness

To ensure exhaustive collection, the list of patients with sarcoma included in the study was checked against different sources: the list of pediatric patients with sarcoma obtained from the Rhone-Alpes children's tumor registry (ARCERRA) [11], the list of patients treated for a sarcoma at the comprehensive cancer centre, the list of patients whose diagnosis was reviewed by the sarcoma expert pathologist of the Comprehensive Cancer Centre (for second opinion) and the list of patients whose management was discussed by the multidisciplinary sarcoma tumor board within the RA region.

### Central pathology review and molecular characterization of the tumors

All suspicious pediatric or adult connective tissue samples were centrally reviewed by the two expert pathologists (DRV, AVD) at the Comprehensive Cancer Center of Lyon to confirm the diagnosis. Immunohistochemical analysis was repeated and completed. Sarcomas were graded according to the grading systems used in the French sarcoma group and to the 2002 WHO classification. When representative material was available for examination, STS were graded using the three-tiered grading system proposed by the Sarcoma Group of the French Federation of Cancer Centers (FNCLCC) [12]. Chondrosarcomas were graded according to the classification of O'Neal and Ackerman, whereas the grade of osteosarcoma (OS) was determined as a function of the subtype (high grade for conventional OS, low grade for parosteal OS). GIST were classified using the risk stratification system published by Fletcher following the international consensus conference [13], and the more recent classification proposed by Miettinen [14].

Soft tissue and bone sarcomas were classified using morphologic and immunohistochemical criteria according to the 2002 WHO classifications [5]. Cutaneous and visceral sarcomas were categorized under three different WHO groups: skin tumors, tumors of the digestive system, and tumors of the breast and female genital organs [15], [16]. In cases where no consensus could be achieved, diagnosis was reviewed collectively by the pathologists of the French sarcoma group.

The molecular characterization of the tumors was established from biopsy or surgical specimens. Specific translocations, gene amplifications and mutations were detected using polymerase chain reaction (PCR), fluorescent in situ hybridization (FISH) and sequencing. Multiplex PCR was also used for analyzing problematic round cell tumors [17].

After central pathology review, all poorly differentiated tumors and sarcomas with strong differential diagnoses and negative molecular results were reviewed again by the national reference pathologists from the French sarcoma group. Decision to finally include or not the cases in the study was based on criteria such as tumor localization and depth.

### Statistical analysis

Statistical analysis was performed with SPSS 12.0 statistical software (SPSS Inc., Chicago, IL). Incidence rates were based on the January 1, 2006 census of Rhone-Alpes obtained from the Institut National de Statistiques Et d'Evaluation (INSEE). Age-adjusted rates were estimated by direct standardization. To allow comparison with previous and future studies, we used data from the French, the European (Scandinavian), the Segi world and the new WHO world standard populations [18].

### Ethics, patient information and follow-up

The study received approval from the French national ethics committee and from the Commission Nationale de l'Informatique et des Libertés (CNIL, protection of individuals with regard to the processing of personal data). This was a descriptive epidemiological study, with no human experimentation and no consequences on patient management; therefore, no institutional review board review was required. Following approval by the French ethics committee, all surgeons in the region received an information letter about the study and were asked to inform their patients with sarcoma that their medical records would be reviewed for the study.

## Results

### Patient characteristics

**Figure 2:**
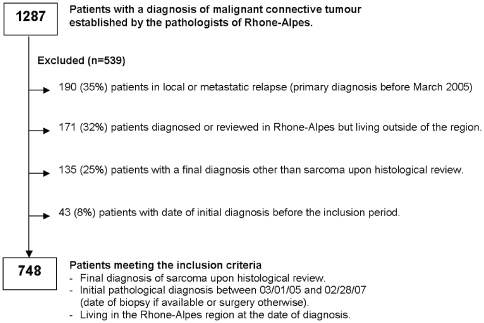
Selection of patients eligible for inclusion.

From March 1, 2005 to February 28, 2006, 1287 suspected sarcoma cases were reported by RA pathologists and 748 (58%) patients were found eligible for inclusion. Figure 2 itemizes the reasons for non inclusion. Twenty-eight private and 15 public pathology laboratories diagnosed respectively 62% (n = 466) and 38% (n = 282) of the cases. The characteristics of the patients are described in table 1. The sex ratio (male/female) was 1.1 and varied with the type of sarcoma. The median age was 60 years, ranging from 0 to 92; 8% (n = 57) of the patients were under 18 years of age and 28% (n = 210) were older than 70 years. The median tumor size was 6 cm for both soft tissue (range = 0.3–40 cm) and visceral (range = 0.3–45 cm) tumors and 7.5 cm for bone tumors (range = 1.3–25 cm). Fifteen tumors (2%) were less than one centimeter in diameter (six GIST, five Kaposi sarcomas, two unclassified sarcomas and two leiomyosarcoma). The tumors were located in all sites of the body (Table 2): 58% (n = 433) in the trunk, 34% (n = 258) in the limbs and 8% (n = 57) in the head and neck. The thigh was the most frequent site for STS and bone sarcomas.

**Table 1:** General characteristics of sarcoma patients in the Rhone-Alpes region, France, 2005–2006.

Characteristic	Total	Years		Type of sarcoma		
		2005	2006	STS	Visceral	Bone
**Total patients**	748 (100%)	378 (100%)	370 (100%)	433 (58%)	237 (32%)	78 (10%)
**Sex**						
Male	391 (52%)	195 (52%)	196 (53%)	245 (57%)	97 (41%)	49 (63%)
Female	357 (48%)	183 (48%)	174 (47%)	188 (43%)	140 (59%)	29 (37%)
**Age at diagnosis (years)**						
Mean	56	56	57	56	61	41
Median	60	60	61	61	62	40
Range	0–92	0–92	0–91	0–92	0–91	6–84
Age group						
0–9	25 (3%)	12 (3%)	13 (3%)	17 (4%)	2 (1%)	6 (8%)
10–19	35 (5%)	18 (5%)	17 (5%)	19 (4%)	3 (1%)	13 (17%)
20–29	39 (5%)	24 (6%)	15 (4%)	20 (5%)	4 (2%)	15 (19%)
30–29	59 (8%)	34 (9%)	25 (7%)	38 (9%)	16 (7%)	5 (6%)
40–49	74 (10%)	40 (11%)	34 (9%)	43 (10%)	25 (10%)	6 (8%)
50–59	133 (18%)	60 (16%)	73 (20%)	70 (16%)	52 (22%)	11(14%)
60–69	161 (21%)	74 (20%)	87 (24%)	99 (23%)	52 (22%)	10 (13%)
70–79	133 (18%)	65 (17%)	68 (18%)	70 (16%)	55 (23%)	8 (10%)
80–89	84 (11%)	47 (12%)	37 (10%)	53 (12%)	27 (11%)	4 (5%)
90+	5 (1%)	4 (1%)	1 (<1%)	4 (1%)	1 (1%)	-
**Tumor size, cm** 1						
≤5	278 (37%)	151 (40%)	127 (34%)	176 (41%)	88 (37%)	14 (18%)
5<≤10	211 (28%)	108 (29%)	103 (28%)	100 (23%)	82 (35%)	29 (37%)
>10	188 (25%)	87 (23%)	101 (27%)	116 (27%)	50 (21%)	22 (28%)
Not available	71 (10%)	32 (8%)	39 (11%)	41 (9%)	17 (7%)	13 (17%)
**Location** 2						
Deep	615 (82%)	297 (79%)	318 (86%)	300 (69%)	237 (100%)	78 (100%)
Superficial	133 (18%)	81 (21%)	52 (14%)	133 (31%)	-	-

1 According to pathology report n = 535 (71%), to imaging n = 133 (18%) or to physical examination n = 9 (1%).

2 Above or under superficial fascia.

**Table 2:** Primary tumor site according to the type of sarcoma (two-year period).

Soft tissue sarcomas(n = 433)		Visceral sarcomas(n = 237)		Bone sarcomas(n = 78)	
Primarytumor site	N (%)	Primarytumor site	N (%)	Primarytumor site	N (%)
**Trunk**	**173 (40)**	**Abdomen**	**161 (68)**	**Limbs**	**43 (55)**
Abdomen	26 (6)	Stomach	85 (36)	Lower limbs	31 (40)
Retroperitoneum	41 (9)	Small intestine	42 (18)	Femur	21 (27)
Thorax	73 (17)	Colon	6 (2)	Tibia	8 (10)
Pelvis	33 (8)	Rectum	4 (2)	Fibula	1 (1)
		Omentum	4 (2)	Metatarsus	1 (1)
**Limbs**	**214 (49)**	Peritoneum	5 (2)	Upper limbs	12 (15)
Lower limbs	155 (36)	Kidney	6 (2)	Humerus	5 (6)
Thigh	88 (20)	Liver	2 (1)	Scapula	3 (4)
Leg	23 (5)	Spleen	2 (1)	Clavicle	2 (2)
Pelvic girdle	23 (5)	Other abdomen	5 (2)	Hand	2 (2)
Foot	8 (2)				
Knee	9 (2)	**Thorax**	**22 (9)**	**Head and Neck**	**10 (13)**
Ankle	4 (1)	Lung	9 (4)	Skull	3(4)
Upper limbs	59 (14)	Pleura	5 (2)	Orbit	2 (2)
Shoulder girdle	22 (5)	Heart	4 (2)	Mandible	1 (1)
Arm	7 (2)	Other Thorax	4 (2)	Other	4 (5)
Forearm	20 (5)				
Elbow	5 (1)	**Pelvis**	**54 (22)**	**Pelvis**	**11 (14)**
Hand	5 (1)	Uterine	42 (18)	**Thorax**	**7 (9)**
		Ovary	3 (1)	Rib	6 (8)
**Head and neck**	**46 (11)**	Bladder	3 (1)	Sternum	1 (1)
		Spermatic cord	3 (1)	**Spine**	**7 (9)**
		Other pelvis	3 (1)		

### Crude and age-adjusted incidence of histological subtypes

Of the 748 sarcoma cases included, 98% (n = 732) were reviewed by the expert pathologists. Review was impossible for the other 16 patients (2%) because there was no tumor tissue available for analysis (three unclassified sarcomas, three angiosarcomas, two leiomyosarcomas, two GIST, two liposarcomas, one DFSP, one Kaposi sarcoma, one osteosarcoma and one epithelioid hemangioendothelioma). Seventy-six percent of the diagnoses (n = 568) were made from surgical specimens and 24% (n = 180) from biopsy samples. Immunohistochemistry was performed whenever necessary, except for two small tumors with limited tissue availability (two unclassified sarcoma).

**Figure 3:**
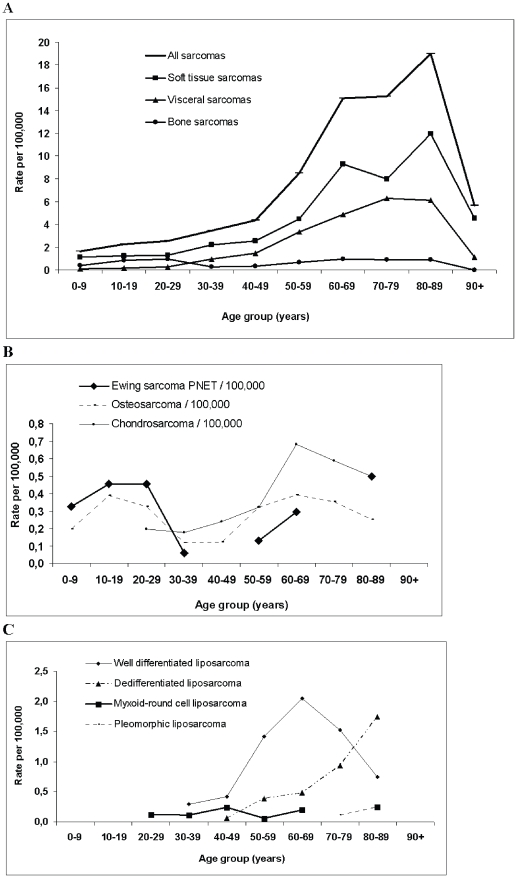
Age-specific incidence rates (per 100,000). (**A**) Age-specific rates by sarcoma type. (**B**) Age-specific rates within bone sarcomas, for the 3 most important histotypes. (**C**) Age-specific rates for liposarcoma subtypes.

Figure 3 shows age-specific incidence rates. The overall sarcoma incidence peaked at 19 per 100,000 in the 80–89 age group (Fig. 3A). Age-specific rates for soft tissue and visceral sarcomas increased regularly up to the oldest age groups. Bone sarcomas presented a biphasic profile with a first peak in young people between 10 and 30 years of age and a second peak in adults between 60 and 90 years of age. Nevertheless this bimodal distribution reflected the combined influence of the three most occurent histotypes (osteosarcoma, chondrosarcoma, Ewing sarcoma), each having a specific distribution (Fig. 3B). Age-specific incidence rates of the different subtypes showed also variation within a histological type (Fig. 3C). The four liposarcomas subtypes showed peaks of incidence in different decades, with myxoid-round cell liposarcoma occurring in patients younger than those affected by dedifferentiated liposarcoma.

Table 3 describes sarcoma incidence rates in the Rhone-Alpes region. The crude and world age standardized incidence rates were respectively 6.2 and 4.8 per 100,000 for the whole sample. Incidence rates for the two histotypes of GIST and unclassified sarcomas reached 1 per 100,000. Fifty-nine percent of visceral sarcomas (140/237) were diagnosed in women, whereas 57% of soft tissue sarcomas (245/433) and 63% of bone sarcomas (49/78) were diagnosed in men.

**Table 3:** Crude and age-standardized incidence rates of the main histological subtypes.

Diagnostic Group	Patients		Crude incidence rates/100,000/year			Age-standardized incidence rates/100,000/year			
	N	(%)	Total	Men	Women	France	Europe	World (Segi)	World (WHO)
**All sarcomas**	748	(100)	6.2	6.7	5.8	6.4	5.6	4.5	4.8
Without Kaposi sarcoma	723	(97)	6.0	6.3	5.7	6.1	5.4	4.4	4.6
**Age group, years**									
Children 0–14	44	(6)	1.9	2.6	1.2	1.9	1.9	1.9	1.9
Adults 15–69	482	(64)	5.8	6.3	5.3	5.9	5.8	5.2	5.1
Elderly 70+	222	(30)	15.8	18.3	14.2	15.8	15.7	15.4	15.7
**Type**									
Soft tissue sarcoma	433	(58)	3.6	4.2	3.0	3.7	3.2	2.6	2.8
Visceral sarcoma	237	(32)	2.0	1.7	2.3	2.0	1.7	1.3	1.4
Bone sarcoma	78	(10)	0.6	0.8	0.5	0.7	0.6	0.6	0.6
**Histological types** 1									
GIST	135	(18)	1.1	1.0	1.2	1.2	0.9	0.7	0.7
Unclassified sarcoma	117	(16)	1.0	1.2	0.8	1.0	0.8	0.6	0.7
Liposarcoma	112	(15)	0.9	1.2	0.7	1.0	0.8	0.6	0.7
Leiomyosarcoma	85	(11)	0.7	0.5	0.9	0.7	0.6	0.5	0.5
Dermatofibrosarcoma	38	(5)	0.3	0.4	0.3	0.3	0.3	0.3	0.3
Osteosarcoma	31	(4)	0.3	0.4	0.2	0.3	0.3	0.3	0.3
Chondrosarcoma	29	(4)	0.2	0.3	0.2	0.2	0.2	0.2	0.2
Ewing sarcoma/PNET	27	(4)	0.2	0.2	0.2	0.2	0.2	0.3	0.3
Rhabdomyosarcoma	26	(3)	0.2	0.3	0.1	0.2	0.2	0.3	0.3
Kaposi sarcoma	25	(3)	0.2	0.4	0.1	0.2	0.2	0.1	0.2
Angiosarcoma	25	(3)	0.2	0.1	0.3	0.2	0.1	0.1	0.1
Myxofibrosarcoma	17	(2)	0.1	0.1	0.1	0.1	0.1	0.1	0.1
Synovial sarcoma	16	(2)	0.1	0.1	0.2	0.1	0.1	0.1	0.1
Endometrial stromal sarcoma	14	(2)	0.1	-	0.2	0.1	0.1	0.1	0.1

1 Histological types with fewer than ten cases per year are not shown.

2006 Rhone-Alpes population: 6,021,352 inhabitants (Male: 2,932,105; Female: 3,089,247).

2006 French population: 61,399,719 inhabitants (Source INSEE).

Four percent (n = 28) of sarcomas occurred in previous radiation fields (13 unclassified sarcomas, 11 angiosarcomas, 3 osteosarcomas, and 1 leiomyosarcoma).

Results of tumor grading and specific risk stratification for GIST are shown in tables 4 and 5. Fifty-six percent (n = 420) of the tumors were graded. Thirty-five percent (n = 147) were classified as low grade, 28% (n = 116) as intermediate grade and 37% (n = 157) as high grade.

**Table 4:** Description of tumor grade.

Histological type	Tumor grade N, (%)								Total
	Low		Intermediate		High		Not done^1^		
**FNCLCC WHO grading**									
Unclassified sarcoma									
Pleomorphic cell sarcoma	2	(4)	13	(24)	28	(51)	12	(22)	55
Spindle cell sarcoma	1	(2)	11	(27)	18	(44)	11	(27)	41
Round cell sarcoma	-	-	-	-	7	(64)	4	(36)	11
Sarcoma not otherwise specified	1	(10)	2	(20)	2	(20)	5	(50)	10
Liposarcoma									
Well differentiated liposarcoma	71	(100)	-	-	-	-	-	-	71
Dedifferentiated liposarcoma	4	(15)	13	(48)	9	(33)	1	(4)	27
Myxoid-round cell liposarcoma	8	(67)	4	(33)	-	-	-	-	12
Pleomorphic liposarcoma	-	-	1	(50)	1	(50)	-	-	2
Non uterine leiomyosarcoma	15	(24)	25	(40)	15	(24)	7	(11)	62
Angiosarcoma	3	(12)	11	(44)	9	(36)	2	(8)	25
Myxofibrosarcoma	2	(12)	8	(47)	7	(41)	-	-	17
Synovial sarcoma									
Monophasic synovial sarcoma	-	-	7	(54)	6	(46)	-	-	13
Biphasic synovial sarcoma	-	-	2	(67)	1	(33)	-	-	3
MPNST	2	(40)	1	(20)	2	(40)	-	-	5
Epithelioid sarcoma	-	-	2	(40)	2	(40)	1	(20)	5
Low grade fibromyxoid sarcoma	4	(100)	-	-	-	-	-	-	4
Fibrosarcoma	1	(33)	-	-	2	(67)	-	-	3
Intimal sarcoma	-	-	-	-	2		-	-	2
Myxoinflammatory fibroblastic sarcoma	2	(100)	-	-	-	-	-	-	2
Composite hemangioendothelioma	1	(100)	-	-	-	-	-	-	1
High-grade phyllodes tumor	-	-	-	-	1	(100)	-	-	1
**Bone sarcoma grading**									
Conventional osteosarcoma	-	-	-	-	26	(100)	-	-	26
Soft tissue osteosarcoma	-	-	1	(34)	2	(66)	-	-	3
Parosteal osteosarcoma	1	(100)	-	-	-	-	-	-	1
Osteosarcoma grade 2	-	-	1	(100)	-	-	-	-	1
Chondrosarcoma	12	(41)	13	(45)	4	(14)	-	-	29
**Uterine sarcoma grading**									
Uterine leiomyosarcoma	3	(13)	1	(4)	10	(44)	9	(39)	23
Endometrial stromal sarcoma	14	(100)	-	-	-	-	-	-	14
Undifferentiated endometrial sarcoma	-	-	-	-	3	(100)	-	-	3

1 Grade was not evaluated due to limited material or too undifferentiated tumor.

**Table 5:** Description of GIST stratification risk.

NIH criteria	Very low risk		Low risk		Intermediate risk		High risk		Not done^1^		Total
GIST	11	(8)	32	(24)	45	(33)	37	(24)	10	(7)	135

AFIP criteria	None risk		Very low risk		Low risk		Moderate risk		High risk		Not done^1^		Total
Gastric	12	(15)	23	(29)	18	(23)	11	(14)	10	(12)	6	(7)	80
Other localization	3	(5)	-	-	10	(18)	14	(26)	26	(47)	2	(4)	55

NIH, National Institutes of Health ; AFIP, Armed Forces Institute of Pathology.

1 Grade was not evaluated due to limited material or too undifferentiated tumor.

### Crude incidence of histological subtypes and molecular subtypes

The total numbers of cases in each histological type and subtype are described in tables 6 and 7, regardless of the primary site (soft tissue, viscera or bone). The four most frequent histotypes represented 60% of all sarcomas. As expected, each histological type had a specific epidemiological profile regarding age and gender; some were more frequent in women (e.g., GIST, leiomyosarcomas and angiosarcomas) and others in men (e.g., unclassified sarcomas, liposarcomas, osteosarcomas, Kaposi sarcomas and rhabdomyosarcomas). Table 8 describes the predominant histotypes for children, adolescents and young adults, adults and the elderly. Most histotypes occurred at any ages, although they are mainly divided in pediatric-type sarcomas occurring in early stage of life and presenting sporadic cases in adults and elderly (e.g. rhabdomyosarcoma, Ewing sarcoma, osteosarcoma) and adult-type sarcomas which emerged in 30- to 49-year-old age group (e.g. GIST, liposarcoma, leiomyosarcoma). Bone sarcoma (osteosarcoma, PNET-Ewing) predominated in adolescents and young adults while dermatofibrosarcoma, Kaposi sarcoma and uterine leiomyosarcoma peaked in 30- to 49-year-old age group. Angiosarcoma affected more frequently elderly mainly due to secondary angiosarcoma (44%) diagnosed after radiation treatment for another cancer. A large proportion of sarcomas (8 to 21%) remained unclassified in each age group.

**Table 6:** Crude incidence of histological subtypes.

Histological types and subtypes	Number				Sex ratio	Age		CIR^1^
	Total	%	2005	2006	(M/F)	Median	Range	
GIST	135	(18)	70	65	0.8	65	(34–91)	1.12
Unclassified sarcoma^2^	117	(16)	57	60	1.4	66	(3–92)	0.97
Pleomorphic cell sarcoma	55	(7)	27	28	1.4	67	(18–91)	0.46
Spindle cell sarcoma	41	(5)	15	26	1.6	67	(27–92)	0.34
Round cell sarcoma	11	(1)	7	4	0.4	25	(3–83)	0.09
Sarcoma not otherwise specified	10	(1)	8	2	4.0	68	(49–84)	0.08
Liposarcoma	112	(15)	56	56	1.7	61	(26–88)	0.93
Well differentiated liposarcoma	71	(9)	36	35	1.8	60	(32–88)	0.59
Dedifferentiated liposarcoma	27	(4)	14	13	1.2	72	(47–84)	0.22
Myxoid-round cell liposarcoma	12	(2)	5	7	2.0	47	(26–81)	0.10
Pleomorphic liposarcoma	2	(<1)	1	1	1.0	78	(72–85)	0.02
Leiomyosarcoma	85	(11)	40	45	0.5	62	(28–87)	0.71
Non uterine leiomyosarcoma	62	(8)	28	34	0.9	62	(28–87)	0.51
Uterine leiomyosarcoma	23	(3)	12	11	-	53	(40–84)	0.20
Dermatofibrosarcoma protuberans	38	(5)	22	16	1.2	37	(8–91)	0.32
Osteosarcoma	31	(4)	19	12	2.1	36	(6–80)	0.26
Conventional osteosarcoma	26	(4)	17	9	3.3	32	(6–80)	0.22
Soft tissue osteosarcoma	3	(<1)	1	2	0.5	60	(30–67)	0.02
Parosteal osteosarcoma	1	(<1)	1	0	-	25	(25)	0.01
Osteosarcoma grade 2	1	(<1)	0	1	-	49	(49)	0.01
Chondrosarcoma	29	(4)	11	18	1.2	59	(20–83)	0.24
Ewing sarcoma/PNET	27	(4)	13	14	0.9	23	(1–83)	0.22
Rhabdomyosarcoma	26	(3)	13	13	3.3	12	(1–83)	0.22
Embryonal rhabdomyosarcoma	12	(2)	5	7	3.0	11	(2–25)	0.10
Alveolar rhabdomyosarcoma	8	(1)	4	4	3.0	7	(1–34)	0.07
Pleomorphic rhabdomyosarcoma	4	(<1)	2	2	3.0	64	(38–82)	0.03
Spindle cell rhabdomyosarcoma	2	(<1)	2	0	-	76	(70–83)	0.02
Kaposi sarcoma	25	(3)	14	11	5.3	59	(30–90)	0.21
Angiosarcoma	25	(3)	13	12	0.5	75	(39–84)	0.21
Myxofibrosarcoma	17	(2)	9	8	0.9	63	(37–84)	0.14
Synovial sarcoma	16	(2)	8	8	0.6	35	(13–87)	0.13
Monophasic synovial sarcoma	13	(2)	7	6	0.4	32	(13–87)	0.11
Biphasic synovial sarcoma	3	(<1)	1	2	2.0	41	(26–43)	0.02
Endometrial stromal sarcoma	14	(2)	6	8	-	49	(23–71)	0.12
Malignant solitary fibrous tumor	8	(1)	3	5	1.7	71	(61–77)	0.07
Other	43	(6)	24	19	1.1	-	-	0.36
**TOTAL**	**748**	**(100)**	**378**	**370**	**1.1**	**60**	**(1–92)**	**6.21**

GIST, gastrointestinal stromal tumor; PNET, primitive neuroectodermal tumor.

1 Crude incidence rate/100,000/year.

2 Unclassified sarcomas were divided in subtypes according to morphological features based on experts' advice.

**Table 7:** Rare histological types of sarcoma.

Histological types	Number				Crude incidence rate/million/year
	Total	%	2005	2006	
MPNST^1^	5	(0.7)	3	2	0.42
Epithelioid sarcoma	5	(0.7)	2	3	0.42
Low grade fibromyxoid sarcoma	4	(0.5)	2	2	0.33
Desmoplastic small round cell tumor	4	(0.5)	3	1	0.33
Undifferentiated endometrial sarcoma	4	(0.5)	2	2	0.33
Rhabdoid tumor	3	(0.4)	2	1	0.25
Epithelioid hemangioendothelioma	3	(0.4)	2	1	0.25
Fibrosarcoma	3	(0.4)	1	2	0.25
Intimal sarcoma	2	(0.3)	2	0	0.17
Malignant inflammatory myofibroblastic tumor	2	(0.3)	2	0	0.17
Myxoinflammatory fibroblastic sarcoma	2	(0.3)	1	1	0.17
PEComa^2^	2	(0.3)	1	1	0.17
Composite hemangioendothelioma	1	(0.1)	1	0	0.08
High grade phyllodes tumor	1	(0.1)	0	1	0.08
Alveolar soft part sarcoma	1	(0.1)	0	1	0.08
Clear cell sarcoma of kidney	1	(0.1)	0	1	0.08

1 MPNST, malignant peripheral nerve sheath tumor.

2 PEComa, neoplasm with perivascular epithelioid cell differentiation.

**Table 8:** Sarcoma types by percentage of cases for age groups.

Age 0–14Children(n = 44 ; 6%)		Age 15–29Adolescents and young adults(n = 55 ; 7%)		Age 30–49Adults(n = 133 ; 18%)		Age 50–69AdultsMiddle-aged(n = 294 ; 39%)		Age 70+Elderly(n = 222 ; 30%)	
Rhabdomyosarcoma	36	Osteosarcoma	20	DFSP	14	GIST	22	GIST	24
PNET/Ewing	20	PNET/Ewing	18	Liposarcoma	14	Liposarcoma	19	Unclass. sarcoma	21
Unclass. sarcoma	9	DFSP	11	GIST	13	Unclass. sarcoma	17	Liposarcoma	15
Osteosarcoma	7	Unclass. sarcoma	11	Unclass. sarcoma	8	Leiomyosarcoma	11	Leiomyosarcoma	10
Rhabdoid tumor	7	Synovial sarcoma	9	Kaposi sarcoma	7	Chondrosarcoma	4	Angiosarcoma	7
DFSP	5	Chondrosarcoma	5	Uterine LMS	6	DFSP	3	Chondrosarcoma	3
Synovial sarcoma	5	Rhabdomyosarcoma	5	Chondrosarcoma	5	Uterine LMS	3	Kaposi sarcoma	3
DSRCT	5	Leiomyosarcoma	4	Leiomyosarcoma	5	Osteosarcoma	3	Myxofibrosarcoma	3
Other	6	Liposarcoma	4	ES sarcoma	5	Kaposi sarcoma	3	Uterine LMS	3
		Epithelioid sarcoma	4	Myxofibrosarcoma	5	ES sarcoma	2	Mal. solit. fibr. tumor	2
		DSRCT	4	Angiosarcoma	3	Angiosarcoma	2	Osteosarcoma	2
		MIM Tumor	4	Osteosarcoma	3	PNET/Ewing	2	Rhabdomyosarcoma	2
		ES sarcoma	2	Synovial sarcoma	3	Synovial sarcoma	1	Other	5
				Other	9	Myxofibrosarcoma	1		
						Other	7		

PNET, primitive neuroectodermal tumor; Unclass. sarcoma, unclassified sarcoma; DFSP, dermatofibrosarcoma protuberans; DSRCT, desmoplastic small round cell tumor; Other, other sarcoma; MIM tumor, malignant inflammatory myofibroblastic tumor; ES sarcoma, endometrial stromal sarcoma; GIST, gastrointestinal stromal tumor; Uterine LMS, uterine leiomyosarcoma; Mal. solit. Fibr. tumor, malignant solitary fibrous tumor.

Of the 748 cases studied, 48% (n = 362) had known molecular alterations: 56% (32/57) of the tumors diagnosed in patients under 19 years of age versus 48% (330/691) in adult patients. Molecular characterization was performed in 85% (n = 306) of these 362 tumors. The remaining 15% (n = 56) could not be analyzed, either because the tumor material had been fixed in Bouin's solution (n = 22), or there was insufficient material for analysis (n = 20) or the technique was not available in France (n = 14). Table 9 presents the distribution and the crude incidence of molecular subtypes in the Rhone-Alpes region. Sarcomas with specific translocations, with point mutations (deletion, insertion, duplication) or with gene amplifications represented respectively 35%, 38% and 27% of all cases with known molecular alterations. Population of GIST was more detailed by Cassier et al. [19].

**Table 9:** Distribution of molecular subtypes for sarcoma with specific gene abnormality (N = 362).

Histological types	No.	Molecular biology analysis performed		Molecular abnormality			
		No. (%)	Results	Type	Involved gene(s)	No. (%)	CIR^1^
			Positive/Negative/Failure				
GIST	135	119 (88)	85/14/20	Mutation	C-kit exon 11	55 (56)	0.46
					C-kit exon 9	10 (10)	0.08
					C-kit exon 13	4 (4)	0.03
					C-kit exon 17	1 (1)	0.01
					PDGFRA exon 18	14 (14)	0.12
					PDGFRA exon 12	1 (1)	0.01
					Wild type	14 (14)	0.12
Well differentiated liposarcoma	71	64 (90)	52/3/9	Amplification	MDM2 and CDK4	42 (80)	0.35
					MDM2 only	9 (18)	0.07
					MDM2 and HMGA2	1 (2)	0.01
Dedifferentiated liposarcoma	27	23 (85)	20/1/2	Amplification	MDM2 and CDK4	19 (95)	0.16
					MDM2 only	1 (5)	0.01
Myxoid/Round cell liposarcoma	12	12 (100)	7/1/4	Fusion transcript	FUS-CHOP	3 (43)	0.02
					Multiplex^4^	3 (43)	0.02
					EWS-CHOP	1 (14)	0.01
Dermatofibrosarcoma protuberans	38	25 (66)	19/2/4	Fusion transcript	COL1A1-PDGFB	19 (100)	0.16
Ewing/PNET	27	26 (96)	23/0/3	Fusion transcript	Multiplex^5^	13 (56)	0.11
					EWSR1-FLI1	8 (35)	0.07
					EWSR1-ERG	2 (9)	0.02
Synovial sarcoma	16	16 (100)	16/0/0	Fusion transcript	SYT-SSX1	9 (56)	0.07
					SYT-SSX2	6 (38)	0.05
					Multiplex	1 (6)	0.01
Endometrial stromal sarcoma^2^	14	0 (0)	-	Fusion transcript	-	-	
Alveolar rhabdomyosarcoma	8	8 (100)	7/1/0	Fusion transcript	PAX3-FKHR	3 (42)	0.02
					PAX7-FKHR	2 (29)	0.02
					Multiplex^6^	2 (29)	0.02
Desmoplastic small round cell tumor	4	4 (100)	4/0/0	Fusion transcript	EWSR1-WT1	4 (100)	0.03
Low grade fibromyxoid sarcoma	4	4 (100)	4/0/0	Fusion transcript	FUS-CREB3L2	4 (100)	0.03
Rhabdoid tumor	3	3 (100)	3/0/0	Mutation	hSNF5-INI1	3 (100)	0.02
Malignant inflammatory myofibroblastic tumor^3^	2	1 (50)	0/0/1	Fusion transcript	-		
Alveolar soft part sarcoma	1	1 (100)	1/0/0	Fusion transcript	ASPL-TFE3	1 (100)	0.01
**Total**	**362**	**306 (84)**	**241/22/43**				

GIST, gastrointestinal stromal tumors; PNET, primitive neuroectodermal tumor.

1 CIR: Crude incidence rate/100,000/year.

2 Molecular biology analysis not performed in France.

3 Detection of rearrangement of ALK gene.

4 Possible fusion transcript: TLS-CHOP; EWS-CHOP.

5 Possible fusion transcript: EWS-FLI1; EWS-ERG; EWS-FEV; EWS-EIAF; EWS-ETV1.

6 Possible fusion transcript: PAX3-FKHR; PAX7-FKHR.

## Discussion

The objective of the present study was to determine the overall incidence of sarcoma as well as the incidence of histological and molecular subtypes in a typical European region of 6 million inhabitants. This involved the collection of all cases diagnosed in the region, as well as the central review and molecular testing of these rare tumors. Patient data collected through a systematic review of patients' medical records were centrally reviewed. To our knowledge, although many studies have reported on the incidence of primary bone and soft tissue lesions, this is the first exhaustive collection of cases on a regional basis, with centralized pathology review coupled with molecular characterization.

The present study identified 748 new cases of sarcoma over a two-year period instead of the 200 per year expected. Of these, 98% were reviewed by regional and national experts in sarcoma pathology, all diagnoses were confirmed by immunohistochemistry, and 85% of cases with molecular alterations could be characterized using molecular techniques. On the basis of these numbers, the French and world age-standardized incidence rates were respectively 6.4 and 4.8 per 100,000 population, which is higher than the rates reported in previous publications, either in the USA or in Europe (between 1 and 3 per 100,000), even though some studies have reported higher results [20], [21]. In children under 15 years of age, the incidence of STS and bone tumors was estimated to be respectively 0.9 and 0.6 per 100,000 per year [22], [23]. In 2005, the number of new cases of cancer of all sites was estimated to 27,869 in the Rhone-Alpes region [24]. With a mean annual rate of 374 new cases in this study, sarcoma thus represents 1.3% of all new cancer cases in the region.

The work presented here differs from previous large retrospective studies which have used a different methodology, and from results published by cancer registries, from which incidence data have been extrapolated. Age-standardized incidence rates of STS are fairly constant in most areas covered by cancer registration, and range from 1–3 per hundred thousand population [25]. However, because of the rarity and heterogeneity of primary sites and presentations of STS, general registries do not provide routine data about their incidence. The main sources of incidence data for sarcoma are the world databases of the International Agency for Research on Cancer (IARC) [25], the American Surveillance, Epidemiology and End Results (SEER) Program [21], [26], the European database of the Automated Childhood Cancer Information System (ACCIS) [27] and the national coverage registry of the Nordic countries [28].

Because of the great number of cases recorded, diagnosis is not always reviewed by pathology experts, which is a serious limitation in the case of sarcomas, given the frequency of misdiagnosis with carcinoma, melanoma or benign tumor, or even between histological subtypes [29], [30]. The reproducibility of sarcoma diagnosis, and particularly of soft tissue sarcoma, is relatively poor across pathologists who are not familiar with these lesions, and the histopathological classification of this cancer in cancer registries is often inconsistent. Epidemiological studies have suffered from this misclassification of histology. A concordance study performed in Rhone-Alpes comparing primary diagnosis and systematic review by expert showed that 46% of diagnoses were modified at second reading and that in 19% of cases there was a discordance in the histological type [31].

In addition, the design of registries is not always suitable for sarcomas. The data collected are incomplete, mainly because of the broad diversity of morphological entities and of the classification of data per anatomic site which does not distinguish visceral sarcomas (e.g., GIST are counted with “digestive cancers”, uterine sarcomas with “uterine cancers”). Very few registry studies have focused and produced data on the histological classification of sarcomas; their results have shown that failing to include tumors arising in specific organs resulted in an underestimation of the overall incidence of sarcoma by 50% [21]. Pediatric registries have reported more accurate data on incidence rates of sarcoma in children and adolescents because the nosologic classifications for children malignancies is primarily based on histology [32], [33] and only few cases of visceral sarcoma arise in children or adolescents (two cases in our study).

Any comparisons with existing datasets may be affected by the specific population under study, by changes in sarcoma incidence over time, and by possible ascertainment biases. We excluded the possibility of an ascertainment bias because of the reputation and experience of the local clinician in the management of sarcoma. The place of residence at the first suspicion of sarcoma was collected for each patient and those who moved to Rhone-Alpes after the first diagnosis of sarcoma were excluded from the analysis. Moreover, our method of case ascertainment is likely to have missed some false negatives (i.e., sarcomas misdiagnosed as other cancer types) and the true incidence of sarcoma incidence might be higher.

Concerning the specific population under study, the overall distribution of sarcoma in Rhone-Alpes is not considered different from the rest of France [34]. Rhone-Alpes is one of the French regions with the youngest population (Source INSEE) though the age demographics are older than in the European and North American reference populations. Ethnic differences might account for some of the variance with published data. The overall incidence of STS is known to be higher among African Americans than among Caucasian patients [9], [21] while Ewing sarcoma showed a striking incidence by race with the great majority of cases occurring in white patients [35]. The Rhone-Alpes population is principally Caucasian, however, no ethnic information was recorded in the study. The bone sarcoma incidence rates reported in the present study and their age-specific distribution were similar to those recently reported in other countries with Caucasian population [36].

The time period under question may also account for the differences with published data. The obvious point of concern is the incidence of Kaposi's sarcoma, which has clearly varied with HIV prevalence over time and across the country. When excluding patients with Kaposi's sarcoma, the crude and world age-standardized incidence rates in our study were respectively 6.0 and 4.6 per 100,000/year. Previous reported incidence rates of soft tissue sarcoma were different amongst countries, even in neighbored European countries, with different distribution of the most common histological subtypes [37]. This was due to different inclusion criteria considering or not sarcoma of intermediate malignancy (e.g. dermatofibrosarcoma), to the small absolute number of cases and to inherent classifications. In the present study, we included GIST (a type of sarcoma with a specific biology) and all histological types of sarcoma described in 4 different WHO groups.

Because of changes and evolutions in the histological classifications of sarcoma, it is not really possible to compare the data collected over the decades. Whether the incidence of sarcoma increases worldwide is unclear: increased rates have been reported, sometimes due to increased incidence of Kaposi sarcoma [20], [38], but such increase was also observed in patients without Kaposi sarcoma [9]. Similar controversial findings are reported in childhood cancer, with some studies reporting increased sarcoma incidence rates [39] not confirmed by others [40]. It is yet unclear whether the incidence of sarcoma increases because of environmental or other behavioral changes, or whether there is only an apparent increase due to modifications in the registration process. Moreover, epidemiological studies are limited by the histopathological misclassification of these rare tumors in cancer registries, both between histotypes (sarcoma vs. other) and among histological subtypes (e.g., leiomyosarcoma vs. other). To test hypotheses regarding risk factors, one must be able to accurately measure disease incidence by age and by histological type. In parallel to the data collected by registries in large patient series, an exhaustive regional study based on morphological criteria is needed.

In addition to describing the overall incidence of sarcoma (all types, all ages), the present study also helps to further refine the estimation of the incidence of the different subsets. GIST was the more frequent histotype reported with a predominance in women, while other published series indicate a more mixed population or even sometimes a male predominance [41]. The incidence of dermatofibrosarcoma protuberans was unexpectedly high (5%) whereas that of synovial sarcoma was lower than expected (2%). In our study, 49% of all STS occurred in the limbs, which is consistent with results of previous site distribution studies. However, if all sarcoma subsets are taken into account, this site distribution changes, with the trunk becoming the primary site of occurrence (58%). The most common histological types in previous series were malignant fibrous histiocytoma (MFH), leiomyosarcoma, liposarcoma and fibrosarcoma. In the present study, the main histological types were GIST, unclassified sarcoma, liposarcoma and leiomyosarcoma. These type distribution differences may be due to recent advances in immunohistochemistry, cytogenetic and molecular biology. With the development of numerous new antibodies since the 1980s, immunohistochemistry has become the most accurate and reproducible tool for sarcoma diagnosis and has provided pathologists with new tests to distinguish between the different histotypes of sarcoma [42]. The distribution of sarcoma subtypes has significantly evolved since the publication of the latest WHO histological classification in 2002 which took into account the results of immunohistochemistry. Two large categories that existed before 2002, MFH and fibrosarcoma, have now become much rarer [43]. Furthermore, the incidence of GIST has increased from 0.2 to more than 1/100,000 in several European countries after the introduction in 2001 of anti-CD117 antibody for immunohistochemical staining [8], [41], [44]. Rates seem to have remained stable since then [44], which tends to prove that the higher number of GIST was due to the change in diagnostic methods and to the reclassification of many mesenchymal gastrointestinal tumors previously diagnosed as smooth-muscle tumors like leiomyosarcomas [45].

The development of molecular biology has also proven essential for the diagnosis of sarcoma subtypes and for the refined classification of sarcomas [46]. This technique is suitable for use in about 50% of sarcomas with specific known genomic abnormality. In addition to clinical presentation and morphology, molecular testing also contributes to distinguishing between malignant and benign tumors (e.g., liposarcomas and lipomas) or between sarcomas of similar morphology (e.g., round cell tumors). Molecular diagnosis has been used for several years in routine for pediatric round cell tumors, specific translocations being used as diagnostic markers.

Molecular biology has a growing impact and in the future, the molecular-based classification of sarcoma should be as important as the classification in histological subtypes. Molecular characterization already has clinical implications for some subtypes of sarcoma, either for prognosis (e.g., poor prognosis for patients with KIT exon 11 deletion in GIST, FKHR-PAX3 expression in metastatic rhabdomyosarcoma and SYT-SSX1 fusion type in synovial sarcoma) [47]–[49], treatment (adjuvant radiotherapy), or response to treatment (e.g. better response to imatinib for GIST with exon 11 mutation than with exon 9 mutation) [50]. In our population-based study, the incidence of PDGFRA mutated GIST was higher than previously reported in patients with advanced disease [50] indicating that tumors bearing mutant PDGFRA have a more indolent behaviour [19]. Moreover, Williamson et al. demonstrated that rather than histology, the key factor in terms of biology and clinical progression of rhabdomyosarcoma was the presence or absence of a fusion gene [51]. The molecular profiling approach used in our study was relatively standard and the technology evolves rapidly [52]. Novel molecular karyotyping techniques, such as array comparative genomic hybridization (aCGH) or gene expression analysis led to improve sarcoma classification by defining tumor-specific clusters that have potential value for resolution of differential diagnoses (e.g. wide separation of GIST from leiomyosarcoma) [53] and led to the identification of new diagnostic markers as well (e.g. DOG1 helpful in recognizing KIT-negative GIST) [54]. Full transcriptome sequencing and other genomic, proteomic and epigenetic profiling approaches should become available in the near future and will allow to better characterize the different histotypes of sarcoma and to better understand their complex genetic structure with numerous rearrangements. Anyway, molecular techniques have yet revealed that sarcomas were different entities with different biologies [54] and have allowed a better understanding of the pathogenesis of some types of sarcomas (e.g. initiating role of mutation of KIT or PDGFRA receptors) [55].

This study was based on the voluntary participation of the different pathology laboratories and the first data source was their spontaneous notifications. All efforts were made to ensure the accuracy of the results and the exhaustiveness of the collection. No list can be totally exhaustive and all offer different levels of quality, but the different cross-checks between lists tend to indicate that we have collected all the sarcoma cases diagnosed in the region during the study period. These good results can be attributed to the effective collaboration between the different specialists and referents.

Soft tissue, visceral and bone sarcoma represent three heterogeneous groups of mesenchymal neoplasms, with different methods of diagnosis, different classifications, different staging and treatment approaches and different management. Nevertheless, we have deliberately collected and grouped all histological types because only this accurate collection can ensure exhaustiveness. For example, the collection of all histological types of bone sarcoma allowed to collect cases of extraskeletal bone sarcoma (e.g. Ewing sarcoma) that would have been missed otherwise.

Sarcomas represent a heterogeneous group of malignancies which may occur at any site and any age. All types occur across the age spectrum [56] but distribution of histotypes during childhood is striking different with what is described in adults or in adolescent and young adult. Specific molecular events defined sarcoma histological types: most pediatric-type sarcoma are associated with translocation due to the high incidence of genetic factors unlike adult-type sarcoma, with more complexe karyotypes, for which environmental factors will tend to influence more often. Adult sarcomas have different prognostic factors [57] and significantly worse outcomes than children sarcomas [56], [58] due to inherent biological differences. Sarcomas present a specific epidemiology per histological and molecular subtypes leading to the conclusion that they comprise multiple aetiologically distinct entities rather than a single disease.

This study allowed to detect and overcome the low frequency of sarcoma. The world age-standardized incidence rate of sarcomas taken as a whole is 5 per 100,000 population per year. This is the first prospective and exhaustive study of sarcoma in Europe, with complete pathological review and updated tumor classification using immunohistochemistry and molecular biology. Our results should prove useful for the development of future targeted treatments since the figures presented here are more accurate than those described in the literature.
